# Comparison of mean pain score using topical and oral metronidazole in post milligan morgan hemorrhoidectomy patient; A randomized controlled trial

**DOI:** 10.12669/pjms.36.5.1796

**Published:** 2020

**Authors:** Syed Tatheer Abbas, Ahmad Raza, Ihtasham Muhammad Ch, Tahira Hameed, Nausheen Hasham, Naveed Arshad

**Affiliations:** 1 Syed Tatheer Abbas, FCPS. Department of Surgery, Akbar Niazi Teaching Hospital, Islamabad, Pakistan; 2 Ahmad Raza, MRCS, FCPS. Department of Surgery, Akbar Niazi Teaching Hospital, Islamabad, Pakistan; 3 Ihtasham Muhammad Ch, FCPS. Associate Professor Department of Surgery, Al-Nafees Medical College, Islamabad, Pakistan; 4 Tahira Hameed, MBBS, MRCS. Registrar, Department of Surgery, Akbar Niazi Teaching Hospital, Islamabad, Pakistan; 5 Nausheen Hasham, MBBS. Registrar, Department of Surgery, Akbar Niazi Teaching Hospital, Islamabad, Pakistan; 6 Naveed Arshad M.Phil. Assistant Professor, Rehabilitation Department, Islamabad Medical and Dental College, Islamabad, Pakistan

**Keywords:** Hemorrhoidectomy, Pain, Metronidazole

## Abstract

**Background and Objectives:**

Milligan Morgan Hemorrhoidectomy is one of the most commonly performed surgeries. Post-operative pain is the major cause of morbidity in post hemorrhoidectomy patients. Metronidazole has an established role in relieving post hemorrhoidectomy pain. The objective was to evaluate the pain score by using topical and oral metronidazole in post Milligan Morgan hemorrhoidectomy patients.

**Methods:**

A prospective randomized controlled trail was done in surgical departments of Akbar Niazi Teaching Hospital, Islamabad. A total of 166 consecutive patients with 3^rd^ and 4^th^ degree hemorrhoids were presenting in surgical OPD and who underwent Milligan Morgan hemorrhoidectomy between July 2018 and December 2018 were included in the study. Patients allocated in two groups, Group-A received topical metronidazole 10% post hemorrhoidectomy for seven days and Group-B were given oral metronidazole 400mg for 07 days. Analgesics were given on patient’s requirement. Patients post hemorrhoidectomy pain was recorded by using VSA scale at baseline (1^st^ day) and on 7^th^ post-operative day. Data analysis was done by using SPSS 21. Pain calculated by compared in terms of mean, standard deviation between groups and independent sample T test was used. Association between demographic details like gender and ages of the patients with pain scale on 7^th^ day was measured and chi-square distribution was used.

**Results:**

Total 166 patients were included in this study. The mean ages of the patients were 44.07±10.62 years with minimum 20 and maximum 60 years. Out of 166 patients, female were 55 (33.1%) and male were 111 (66.9%). Results showed significantly lower pain in patients using topical metronidazole as compared to oral metronidazole (*p=0.001*). Association of pain with respect to ages was insignificant (*p=0.202*) whereas between genders inside the groups showed significant difference (*p=0.028*).

**Conclusion:**

These results showed that topical metronidazole significantly reduces pain in post hemorrhoidectomy patients as compared to oral metronidazole overall and even when data stratified among age, gender and type of hemorrhoids.

## INTRODUCTION

Hemorrhoids are symptomatic and prolapsed anal cushions at 3, 7, 11 o’clock. Secondary hemorrhoids are between them. Patients present with complaints of fresh bleeding per rectum, mucous discharge, pain rectal discomfort and swelling. There are four degrees of hemorrhoids. In 1^st^ degree there is bleeding per rectum with no prolapse, in 2^nd^ degree there is prolapse with spontaneous reduction, 3^rd^ degree hemorrhoids are manually reduced, and 4^th^ degree are completely prolapse with no reduction.^1^,^2^

Hemorrhoids not responding to non-operative management, recurrent after banding and sclerotherapy and 3^rd^ and 4^th^ degree are treated surgically by hemorrhoidectomy, stapler hemorrhoidopexy.^2^ Milligan Morgan hemorrhoidectomy is still gold standard treatment for hemorrhoids and commonly performed surgery.^2^ But it is associated with post-operative complications such as pain, bleeding, non-healing wound, incontinence, stenosis and urinary retention. Pain is the major post-operative complaint and is attributed to surgical wound in sensitive anoderm, edema, spasm and infection.^3^ Various remedies have been suggested to alleviate the post-operative pain like GTN 0.2%, topical NSAIDS, Ca channel blockers and metronidazole.^4^ Studies show that metronidazole in both forms (topical and oral) significantly reduces the post-operative pain (*p0.004, p0.0011*)^5^,^6^ and improves the wound healing compared to placebo.

The rationale behind doing this study was that limited data was available regarding the use of topical metronidazole for post hemorrhoidectomy pain. And topical metronidazole has less systemic side effects so the patient’s compliance was improved. Topical metronidazole is not frequently used in our setup. So this study provided us with fresh first hand and local evidence about mean pain score by using topical and oral metronidazole. The objectives of this study are to investigate and compared whether mean pain score after topical metronidazole 10% was equal or less than oral metronidazole.

## METHODS

After approval from ethical committee (Ref. No. 14/ANTH/IRB-2018 dated March 9, 2018) of the hospital, this prospective randomized controlled trial was carried out in surgical department of Akbar Niazi Teaching Hospital (ANTH) from July 2018 to December 2018. After informed written consent a total of 166 patients (sample size calculated by using Mean±SD 3.15±0.8 and 2.8±0.75 with 95% CI)^7^ with 3^rd^, 4^th^ degree hemorrhoids and age 20 to 60 years presenting in OPD were admitted for Milligan Morgan hemorrhoidectomy to be performed by consultant with 05 years’ experience. Patients with diabetes mellitus, chronic liver disease and ASA III, IV were excluded from study. Patients with mental incompetence and patients refusing informed consent were also excluded from study. 83 Patients were randomly allocated to Group-A (Topical Metronidazole) and 83 to Group-B (Oral Metronidazole) by non-probability consecutive sampling. Two groups were made by computer to received either topical metronidazole or oral metronidazole. Randomization numbers were placed inside sequentially numbered opaque envelopes.

The following procedure was done for evaluation of the patients; patients were familiarized with visual analogue scale and test reading was taken preoperatively. At baseline (1^st^ post-operative day) and on 7^th^ post-operative day pain was measured and the score of VAS was calculated. After surgery pain is aggravated due to incisions and hemorrhoidectomy. All patients received their first dose of infusion metronidazole preoperatively. Patients in Group-A received topical metronidazole 10%, 06 hours postoperatively then 08 hourly for 07 days and Group-B received oral metronidazole 400mg, 08 hourly for 07 days. Patients in both groups were advised sitz bath for 15 minutes three times a day and analgesics advised on patient’s requirement. Patient discharged next day. Pain on defecation and during preceding 24 hours was recorded in both groups by using visual analogue scale VAS in centimeters as a single value on 7th post-operative day (follow up visit).

Data analysis was done by SPSS-21. Quantitative variable pain was calculated in terms of mean and standard deviation. Frequency and percentages were calculated for gender and type of hemorrhoids. Mean pain score at baseline and on 7th post-operative day between two groups was compared by using independent sample *t* test keeping p value ≤0.05 as significant. Mean post-operative pain score was stratified among age, gender and type of hemorrhoids to see effect modification, chi-square was used. Results were presented in the form of table and graphs.

**Graph I & II:**
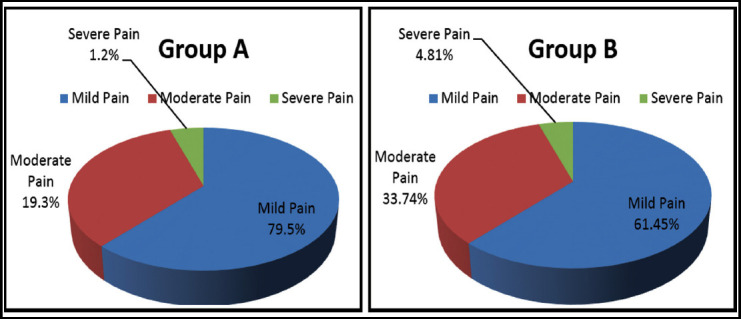
Percentages of post-operative pain within two groups, N = 166.

## RESULTS

Total 166 patients (83 in each group) were evaluated for post hemorrhoidectomy. Mean age in Group-A was 44±10.45 and mean age in Group-B was 43±10.84. Out of 83 patients in Group-A 55 (66.27%) were male and 28 (33.73%) were female. In Group-B male and female ratios were 56 (67.47%), 27 (32.53%) respectively.

Out of 83 patients in Group-A 66 (79.5%) had 3^rd^ degree hemorrhoids and 17 (20.5%) 4^th^ degree hemorrhoids. In Group-B 68 (81.9%) out 83 patients were with 3^rd^ degree and 15 (18.1%) with 4^th^ degree hemorrhoids. The pain range in 3^rd^ and 4^th^ degree hemorrhoids were 5-7 and 6-7 on 1^st^ operative day and 3-5 & 4-6 on 7^th^ operative day on VAS scale respectively.

Pain score at 1^st^ day before surgery and at 7^th^ day after surgery, pre and post interventions were applied of topical and oral metronidazole compared between two groups (Table-I). VAS score on 7^th^ day was compared with gender and ages of the patients (Table-II).

**Table-I:** Comparison pain score at baseline and post intervention between groups; N = 166.

VAS score	Topical metronidazole Group-A	Oral metronidazole Group-B	t*	P-value

	Mean±SD	Mean±SD		
Pain scale at baseline (1st day)	6.01±1.34	6.18±1.68	-0.716	.475
Post intervention of pain scale at (7th day)	3.04±1.41	4.05±1.92	-3.873	.001

* :Independent sample t-test.

**Table-II:** Association VAS on 7th day with gender and ages of patients, N = 166.

		VAS Score on 7th Day					

		Mild	Moderate	Severe	Total	Chi-square value	P-value
Gender	Female	45	10	0	55	15.70	.028
	Male	72	34	5	111		
Total		117	44	5	166		
Ages	20-30 years	16	4	1	21	292.30	.202
	31-40 years	32	6	0	38		
	41-50 years	43	14	1	58		
	51-60 years	26	20	3	49		

Total		117	44	5	166		

Alpha chi-square values have expected count which was less than 5%.

Status of post-operative pain among two groups was analyzed as in Group-A 66 patients had mild pain, 16 had moderate pain, 01 had severe pain. Whereas in Group-B 51 patients had mild pain, 28 had moderate pain and four had severe pain.

## DISCUSSION

Hemorrhoidectomy is one of the most common surgeries performed in our setup and metronidazole is commonly used drug. Milligan Morgan hemorrhoidectomy is the gold standard.^1^,^2^ But it is associated with significant postoperative pain, caused by wound in sensitive anoderm, edema, and infection esp. anaerobic.^3^

This study showed that in Group-A mean pain was 3.04±1.41 on 7^th^ post-operative day. Whereas in Group-B mean pain was 4.05±1.92 (p = 0.001), so there was a significant reduction in pain on 7^th^ postoperative day. Stratification of mean pain with respect to ages was insignificant whereas between genders inside the groups showed significant difference.

The reasons of topical metronidazole were local effects and quick action with better compliance as compared to oral metronidazole with delayed onset and decreased drug provision due to first pass effect while direct vascular permeability and access locally for topical metronidazole at the site of application. Looking at the above statistics and results of this study, we strongly advocate to use topical metronidazole as it improved the patients post-operative pain and reduced morbidity. In our country it is not widely used too.

Studies show that metronidazole reduces postoperative pain^7^-^13^ and improves wound healing^12^ as compared to placebo. Oral metronidazole has systemic side effects as nausea, anorexia and metallic taste.^8^ Studies on topical metronidazole showed that it significantly reduces post-operative pain and has less systemic side effects.^8^,^10^-^13^

The study by Neogi P et al. indicates that metronidazole (oral and topical) significantly reduces postoperative pain on day 3, 7 but showed no difference in between oral and topical metronidazole.^7^ Whereas our study showed significant difference in between the two (topical and oral metronidazole). Ala S et al. used topical metronidazole 10% in post hemorrhoidectomy patients that resulted in significantly reduced pain on day 07 and 14 (*p≤0.04*) as compared to placebo.^6^ Similar results obtained by Gonzalez A et al. in 2015 where patients experienced significantly less post-operative pain.^5^ Pourghassem J et al. in 2012 studied 40 patients randomly distributed between topical metronidazole and placebo, showed that patients on topical metronidazole experienced less pain, they required few analgesics.^13^ The efficacy is thought to be due to bactericidal action and its less understood anti-inflammatory effects. It is also believed that it reduces sphincter spasm. Bijur P et al. showed that the VAS was a highly reliable instrument for measurement of acute pain. Ninety percent of paired measurements, differences between scores obtained one minute apart were 9 mm or less.^14^ Overall the results show that topical metronidazole significantly reduces pain as compared to oral metronidazole.

### Limitations of the study

More male patients (66.9%) were included than females (33.1%). This is because of the social norms where female are reluctant to talk about their disease. Topical metronidazole 10% ointment was not available in our setup and had to be prepared by a local pharmacist.

## CONCLUSION

The study concluded that mean pain score after topical metronidazole is less than oral metronidazole in post Milligan Morgan hemorrhoidectomy patient.

### Author’s Contribution

**STA** provided concept/research design and did data collection, subjects & editing of manuscript.

**AR, NA** did statistical analysis and manuscript writing.

**IC** did editing of manuscript and project management.

**TH, NH** did data collection, subjects and provision of facilities/equipment.

**AR, NA** takes the responsibility and is accountable for all aspects of the work in ensuring that questions related to the accuracy or integrity of any part of the work are appropriately investigated and resolved.
